# NMR and LC-MS-Based Metabolomics to Study Osmotic Stress in Lignan-Deficient Flax

**DOI:** 10.3390/molecules26030767

**Published:** 2021-02-02

**Authors:** Kamar Hamade, Ophélie Fliniaux, Jean-Xavier Fontaine, Roland Molinié, Elvis Otogo Nnang, Solène Bassard, Stéphanie Guénin, Laurent Gutierrez, Eric Lainé, Christophe Hano, Serge Pilard, Akram Hijazi, Assem El Kak, François Mesnard

**Affiliations:** 1 UMRT INRAE 1158 BioEcoAgro, Laboratoire BIOPI, University of Picardie Jules Verne, 80000 Amiens, France; kamar.hamade@u-picardie.fr (K.H.); ophelie.fliniaux@u-picardie.fr (O.F.); jean-xavier.fontaine@u-picardie.fr (J.-X.F.); roland.molinie@u-picardie.fr (R.M.); elvisotogonnang@gmail.com (E.O.N.); solene.bassard@u-picardie.fr (S.B.); 2 CRRBM, University of Picardie Jules Verne, 80000 Amiens, France; stephanie.vandecasteele@u-picardie.fr (S.G.); laurent.gutierrez@u-picardie.fr (L.G.); 3 USC INRAE 1328, Laboratoire LBLGC, Antenne Scientifique Universitaire de Chartres, University of Orleans, 28000 Chartres, France; eric.laine@univ-orleans.fr (E.L.); christophe.hano@univ-orleans.fr (C.H.); 4 Plateforme Analytique, University of Picardie Jules Verne, 80000 Amiens, France; serge.pilard@u-picardie.fr; 5 Platform for Research and Analysis in Environmental Sciences (PRASE), Lebanese University, Beirut 6573, Lebanon; akram.hijazi@ul.edu.lb; 6 Laboratoire de Biotechnologie des Substances Naturelles et Produits de Santé (BSNPS), Lebanese University, Beirut 6573, Lebanon; aelkak@ul.edu.lb

**Keywords:** lignans, secoisolariciresinol, RNA interference, flax, osmotic stress, metabolomics, ^1^H-NMR, LC-MS

## Abstract

Lignans, phenolic plant secondary metabolites, are derived from the phenylpropanoid biosynthetic pathway. Although, being investigated for their health benefits in terms of antioxidant, antitumor, anti-inflammatory and antiviral properties, the role of these molecules in plants remains incompletely elucidated; a potential role in stress response mechanisms has been, however, proposed. In this study, a non-targeted metabolomic analysis of the roots, stems, and leaves of wild-type and PLR1-RNAi transgenic flax, devoid of (+) secoisolariciresinol diglucoside ((+) SDG)—the main flaxseed lignan, was performed using ^1^H-NMR and LC-MS, in order to obtain further insight into the involvement of lignan in the response of plant to osmotic stress. Results showed that wild-type and lignan-deficient flax plants have different metabolic responses after being exposed to osmotic stress conditions, but they both showed the capacity to induce an adaptive response to osmotic stress. These findings suggest the indirect involvement of lignans in osmotic stress response.

## 1. Introduction

Flax (*Linum usitatissimum*) has been extensively used as a plant model for lignan biosynthesis studies. Flaxseed is a rich source of lignans and neolignans and contains about 75–800 times more lignans than other plant-derived food [1]. Secoisolariciresinol diglucoside (SDG) is the major lignan present in flaxseed and exists as a macromolecule in the flaxseed hull. This polymer complex is composed of SDG, which are held together by 3-hydroxy-3-methylglutaric acid (HMGA) residues; The hydroxycinnamic acids, 4-*O*-β-d-glucopyranosyl-*p*-coumaric glucoside (CouAG), and 4-*O*-β-d-glucopyranosyl ferulic acid glucoside (FAG) are ester-linked to the glucosyl moiety of SDG as end units. Stereochemistry and enantiomeric composition of lignans vary between plant species, but also between different organs and/or different developmental stages within the same species [2]. For example, flax seeds contain almost 99% (+)- and 1% (−)-SDG, whereas its aerial parts during flowering stage accumulate only (−)-enantiomers of lignans [3].

In flax, the lignan biosynthesis pathway starts with the stereospecific coupling of two coniferyl alcohol residues, in the presence of dirigent proteins (DIR), leading to the formation of (−)-pinoresinol, which is step-wisely converted into (−)-lariciresinol and then into (+)-secoisolariciresinol through the action of a NADPH-dependent pinoresinol-lariciresinol reductase (PLR). In *L. usitatissimum,* five PLR genes are present in the genome that encodes full PLR proteins, but only two have been widely studied: *LuPLR1* and *LuPLR2*. Both *LuPLR1* and *LuPLR2* transcripts were detected in the seeds and roots but only the *LuPLR2* transcript is detected in leaves and stems [4,5]. PLR1 and PLR2 showed opposite enantiospecificity towards pinoresinol and lariciresinol. Further on, PLR1 catalyzes the conversion of (−)-pinoresinol into (−)-lariciresinol and contributes to the synthesis of (+)-secoisolariciresinol, whereas PLR2 catalyzes the conversion of (+)-pinoresinol into (+)-lariciresinol and contribute to the synthesis of (−)-secoisolariciresinol [5,6].

Lignans are well recognized for their antioxidative, anti-inflammatory, and antiestrogenic activities, key properties that contribute to their ability to reduce risk and protect against various types of cancer, especially hormone-dependent cancers, like breast, colon, and prostate cancer [7,8,9]. Lignans also exhibit an important role in the reduction of hypercholesterolemia, atherosclerosis, hypertension and diabetes [10,11,12,13]. Furthermore, in the last years, several research works demonstrated that dietary lignans could have beneficial effects in the prevention of neurological disorders [14].

However, in plants, the exact biological role of the lignans still remains unclear. They have potent defensive properties, including antiviral [15], antimicrobial [16], antifungal [17,18], and antifeedant activities [19]. Along with these, lignans can act as phytoalexins: they are synthesized in various plant species in response to fungal infections [20]. A possible phytoanticipin activity of lignans is also reported in heartwood where they act as a protective post-lignification infusion [21]. In addition, due to their antioxidant activity, lignans can protect the seed oil against oxidation by scavenging reactive oxygen species (ROS) or maintaining seed dormancy [22,23,24]. Lignans have been proposed to have a role in defense against oxidative stress during the process of lignification as it is the case in poplar [25]. Additionally, a study by Corbin et al. [26] has shown a regulation of the transcription of the *LuPLR1* gene in flax by the hormone abscisic acid (ABA) suggesting a role of lignan in stress response.

In a previous study, Renouard et al. [27], successfully used the RNAi-mediated gene silencing approach to knock-down the *LuPLR1* gene in flax. As a consequence, the accumulation of the major lignan SDG was nearly suppressed in PLR1-RNAi seeds. These plants, devoid of lignan, provide a tool to allow a comparison between wild-type and PLR1-RNAi transgenic plants, under stress condition and give better insight into the role of lignan in plants.

In the present work, we used the PLR1-RNAi transgenic flax to elucidate whether lignan is involved in the response to osmotic stress (simulating drought stress). For this purpose, we performed a comparative non-targeted metabolic analysis of the roots, stems, and leaves of wild-type and PLR1-RNAi transgenic *L. usitatissimum*, using proton-nuclear magnetic resonance spectroscopy (^1^H-NMR) and liquid chromatography-mass spectrometry (LC-MS), to identify the common and specific metabolic responses in plants under osmotic stress conditions induced by polyethylene glycol (PEG-6000).

## 2. Results and Discussion

### 2.1. Phenotype Analysis

**Figure 1:**
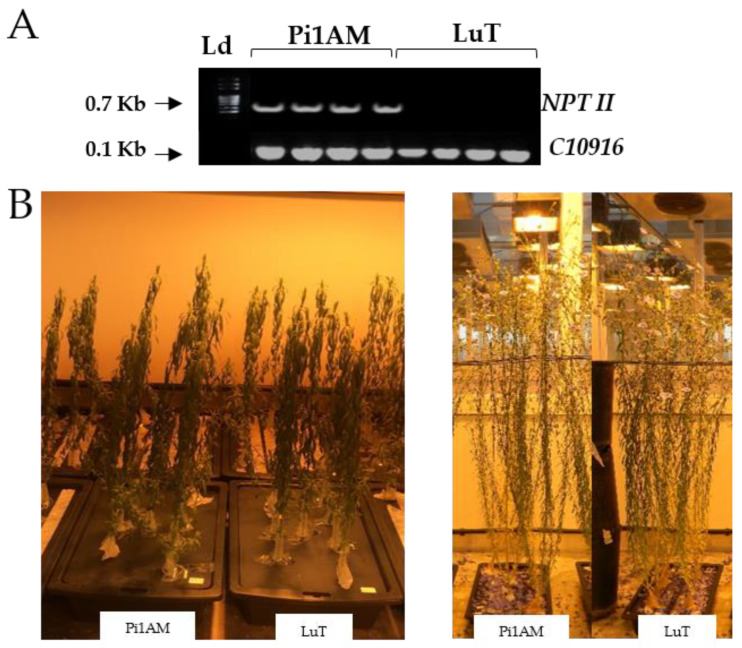
(**A**) Amplification of *NPTII* sequence from genomic DNA of wild-type (LuT) and transgenic plants transformed with pART-RNAiPLR binary vector (Pi1AM). PCR products obtained with a specific set of primers designed for the amplification of a 0.7 Kb *NPTII* fragment were separated by 2.0% agarose gel electrophoresis and visualized with GelRed, under UV light. Ld, a 100 pb DNA Ladder (Solis BioDyne). Flax housekeeping gene was used as a reference gene to normalize the PCR data. (**B**) Phenotypes of wild-type and transgenic plants under control conditions. Pictures were taken at 25 and 75 days after sowing (left and right respectively).

The presence of the selectable marker gene *NPTII* was confirmed in the samples of all homozygous Pi1AM plants selected for the study. Figure 1A shows the PCR analysis of genomic DNA of wild-type (LuT) and transgenic plants (Pi1AM). Plants expressing the *NPTII* marker gene showed no significant morphological differences from the non-transgenic plants, when they were grown under control conditions (Figure 1B). In addition, after harvest, root, stem, and leaf dry weights were determined, and no significant differences were shown between wild-type and PLR1-RNAi transgenic lines (Supplementary Data 1). These observations suggest that the lignans have no essential role in plant growth under the present conditions.

### 2.2. Metabolite Profiling

#### 2.2.1. Difference of Metabolic Profiles between Wild-Type and PLR1-RNAi Transgenic Lines under Control Conditions

Before studying the impact of osmotic stress on the *L. usitatissimum* metabolome, we firstly compared the metabolite composition of each of the three parts (root, stem, and leaf), of wild-type (LuT) and transgenic (Pi1AM) plants, in control conditions at different time points (day 1 (D1) and day 5 (D5)).

Metabolomic profiles analysis was performed using LC-MS and NMR. While NMR is less sensitive than LC-MS, it can be used for quantification and structural identification of unknown compounds. NMR is, therefore, ideal for untargeted metabolomic studies. However, LC-MS is more sensitive than NMR, and can detect a higher number of metabolites, particularly less abundant compounds such as secondary metabolites. Thus, applying both NMR and LC-MS improves the coverage of the metabolome and enhances both metabolite resolution and annotation.

**Figure 2:**
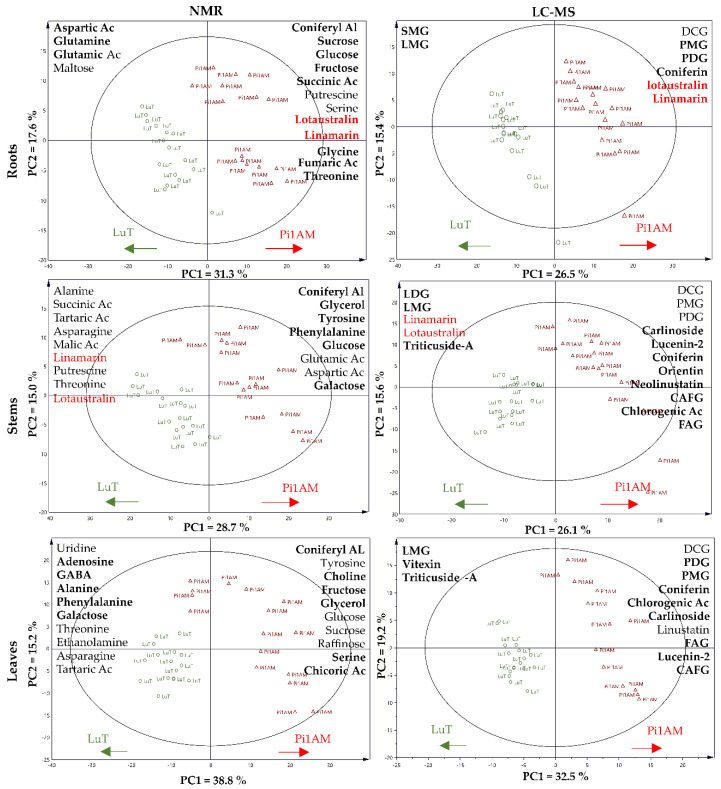
Score plot of partial least squares discriminant analysis (PLS-DA) of metabolite content in flax roots, stems, and leaves, based on ^1^H-NMR and LC-MS data for LuT and Pi1AM, in control conditions. Samples represent LuT plants (circle) and Pi1AM plants (triangle). Arrows indicate the increases in relative metabolite content in various parts of the wild-type (LuT) or transgenic (Pi1AM) plants. Metabolites are listed in descending order of discrimination. Boldface text indicates discriminant metabolites of the two lines in both control and stress conditions. Red text indicates metabolites measured in common with LC-MS and ^1^H-NMR. Variable Importance in Projection (VIP) score and the Log10 (Ratio Pi1AM/LuT) of the main discriminant metabolites for each PLS-DA model, are shown in supplementary data 2.

The LC-MS and NMR spectral data obtained for each plant part, were subjected separately to a multivariate non-targeted analysis-PLS-DA. The score plot of PLS-DA, shown in Figure 2, shows that transgenic LuPLR1-RNAi samples (Pi1AM) were grouped away from the wild-type samples, in various plant parts. These results highlight the metabolomic variability between LuT and Pi1AM. The next step to compare LuT and Pi1AM consisted of identifying the discriminant metabolites in each plant part.

The results of statistical analysis (Wilcoxon Rank Sum test) indicate a significant difference in 22 metabolites in roots, 32 in stems, and 33 in leaves, between LuT and Pi1AM. Differences were observed in primary metabolites as well as in secondary metabolites in different plant parts.

##### Changes in Primary Metabolism

The changes in primary metabolites are more evident by the PLS-DA model obtained with NMR spectral data. Differences in the accumulation of these metabolites between LuT and Pi1AM in different plant parts indicate that the change is not restricted to a few chemical classes or categories, and involves different metabolic pathways.

Among primary metabolites, coniferyl alcohol displayed similar trends in different plant parts: The amount of this compound although, exhibited to be 7.91, 8.65, and 10.32 times higher in Pi1AM than in LuT in roots, stems and leaves, respectively.

In addition, when compared to LuT roots, those of Pi1AM, the transgenic line, had significantly higher contents of nine metabolites but significantly lower contents of four metabolites. These up-accumulated metabolites were mainly glucose, fructose, succinic acid, putrescine, serine, sucrose, glycine, fumaric acid and threonine. In contrast, LuT roots were significantly higher in aspartic acid, glutamine, glutamic acid, and maltose contents.

Moreover, Pi1AM stems presented an up-accumulation of seven metabolites, which include glycerol, tyrosine, phenylalanine, glucose, glutamic acid, aspartic acid, and galactose, when compared to LuT stems while these latter showed an up-accumulation of seven different metabolites identified as alanine, succinic acid, tartaric acid, asparagine, malic acid, putrescine, and threonine.

In leaves, the Pi1AM line had significantly higher contents of nine metabolites than LuT including many sugars such as fructose, glucose, sucrose, raffinose, but also tyrosine, choline, glycerol, serine, and chicoric acid. Despite, LuT displayed a higher content in 10 metabolites including amino acids such as GABA, alanine, phenylalanine, threonine, asparagine, and other compounds, such as uridine, adenosine, galactose, ethanolamine, and tartaric acid.

Despite the genetic modification is in a part of the genome coding for compounds that are involved in metabolic pathways away from the biosynthetic pathway of amino acids, sugars, and organic acids, we observed major changes for these latter. These primary metabolites are central molecules. Changes at these levels can therefore affect a large part of the other metabolic pathways of the plant.

In the different plant parts, nitrogen metabolism showed changes in the content of amino acids such as glutamic acid, glutamine, aspartic acid, and asparagine, in the transgenic line compared to the wild-type. These amino acids are commonly involved in nitrogen recycling and transport.

These observations lead to hypothesize that these amino acids can be affected by the disruption of the activity of phenylalanine ammonia-lyase (PAL) which can be caused by the accumulation of coniferyl alcohol, in the transgenic line.

PAL constitutes the point of connection between the primary metabolism of shikimate, which leads to aromatic amino acids and the secondary metabolism of phenylpropanoids. Generally, phenylpropanoid biosynthesis begins with the deamination of phenylalanine by phenylalanine ammonia-lyase (PAL) to yield cinnamic acid [28]. In the second step, the action of cinnamate 4-hydrolase (C4H) transforms the cinnamic acid into *p*-coumaric acid. Then, 4-coumaroyl CoA ligase (4CL) catalyzes *p*-coumaric acid into *p*-coumaroyl-CoA. This latter is a crucial branch point leading to the generation of various phenolic compounds such as flavonoids, lignins and lignans [29]. In transgenic plants, coniferyl alcohol can accumulate eventually to levels that could be toxic to the plant cells. Therefore, cells can put in place a negative feedback to limit this accumulation. This regulation must act on the pathway of phenylpropanoid, responsible for the production of coniferyl alcohol. PAL are the most potential candidates for this process, since the regulation of PALs can also affect nitrogen metabolism.

In fact, PALs are responsible for the release of NH^4+^ from phenylalanine which is then incorporated into glutamate to form glutamine. Then, amino and amide groups from glutamate and glutamine are transferred to other molecules by transamination and transamidation reactions for the synthesis of amino acids [30].

##### Changes in Secondary Metabolism

On the other hand, and with regard to secondary metabolites, the loading plots obtained from LC-MS data indicate that, the major change can be observed in the lignan metabolite group in the different plant parts. An 18.1, 5.31, and 13.78-fold increase in the content of pinoresinol mono-glucoside (PMG), was observed in roots, stems, and leaves of the Pi1AM line, respectively. The diglucosylated forms of this metabolite (PDG), also showed a 12.27, 4.73 and approximately 15-folds increase in roots, stems, and leaves of Pi1AM, respectively. The drastic accumulation of pinoresinol may initiate a downregulation on the dimerization process of coniferyl alcohol that yield pinoresinol, as a negative feedback, and that, as a consequence, can explain the coniferyl alcohol accumulation in the transgenic line.

However, the accumulation level of pinoresinol in both its mono and diglucoside forms, was higher than that of the coniferyl alcohol, in the transgenic line. This observation suggests that coniferyl alcohol can be accumulated in a different form other than its free form. This hypothesis was supported by an accumulation of coniferin observed in the three parts of the transgenic plant compared to the wild-type.

Coniferin is a glycoside of coniferyl alcohol, and might serve as an alternative precursor in lignan production. Coniferin is accumulated in Pi1AM compared to the LuT line, with 2.32, 2.4, and 7.68-fold increases in roots, stems, and leaves, respectively. This accumulation is also in accordance with the increase of coniferyl alcohol. Thus, it is likely that, the coniferyl alcohol accumulates in both free and glycosylated forms in the transgenic line.

Lariciresinol mono-glucoside (LMG) showed to be lower in Pi1AM when compared to the LuT line, with a Pi1AM/LuT ratios (calculated by the formula: ratio Pi1AM/LuT = content Pi1AM plant/content LuT plant) of 0.31, 0.73, and 0.50 for roots, stems, and leaves, respectively.

In our study, lariciresinol in di-glycosilated form (LDG) is not detected, both in roots and leaves of *L. usitatissimum.* However, LDG is detected in stems in a lower amount in Pi1AM when compared to the LuT (Pi1AM/LuT ratio: 0.17)

The negative correlation between the accumulation of pinoresinol and lariciresinol with the PLR activity confirmed that the accumulation of theses lignans occurred due to the reduction of the PLR enzyme activity.

A remarkable reduction of secoisolariciresinol mono-glucoside (SMG) was observed only in roots of the Pi1AM line compared with that in roots of LuT. Secoisolariciresinol are usually found in a glycosylated form (SDG), its intermediate monoglucoside (SMG) forms not being accumulated in the seed. In this study, the presence of SMG was detected in roots. The decreased amount of SMG in the transgenic line is then, a consequence of the PLR enzyme suppression. PLR, in fact, a bifunctional enzyme which catalyze the reduction of pinoresinol to lariciresinol and then lariciresinol to secoisolaricirésinol [27]. By suppression of *LuPLR1* expression, the lignan biosynthesis pathway from pinoresinol to the end products would be blocked, leading to an accumulation of pinoresinol and a decrease of both lariciresinol and secoisolariciresinol within the transgenic line.

Both PLR1 and PLR2 enzymes were detected in the seeds and roots. However, in stems and leaves, only PLR2 was expressed. However, our results showed that the integration of PLR1-RNAi in the *L. usitatissimum* genome leads to a different metabolome in roots, but also in stems and leaves of wild-type and transgenic lines. Thus, it could be suggested that the suppression of *LuPLR1* expression is accompanied by the *LuPLR2* cosuppression. This can be theoretically, possible due to a major similarity of *LuPLR1* sequence used to synthetize PLR1-RNAi construct, with *LuPLR2* gene (60.3% of sequence homology) as already observed by Corbin et al. [6].

Herein it should be noted that, the decreased rate in both LMG and SMG is not proportionally equivalent to the increased rate of pinoresinol, in the transgenic line. This led us to hypothesize that, in the transgenic line, a compensation can occur with an increase in the production of pinoresinol in order to recover the loss of LMG and SMG. This compensation could be carried out by the cell to maintain the role of lignan.

Because lignans play an important role in plant defense, their content should be maintained in the transgenic line. The low amounts of larciresinol in the cell can therefore activate regulators in order to transmit a signal into the cell to continue producing pinoresinol. A possible regulation of dirigent proteins by abscisic acid ABA could be suggested, since this phytohormone appears to be involved in the regulation of lignan biosynthesis as previously described in *L. usitatissimum* [31], as well as in the regulation of dirigent proteins in response to abiotic stress as previously described in *B. hygrometrica* [32].

Furthermore, the presence of neolignan dehydrodiconiferyl alcohol glucoside (DCG) was detected in roots, stems and leaves of the transgenic lines, but it was not found in the wild-type plant. A similar result was seen in PLR1-RNAi transgenic flax seeds [27]. The aforementioned results can be attributed to a down-regulation of the *LuPLR1* gene expression, leading to the synthesis of the neolignans. Thus, the DCG formation could result from the coupling of two monolignol moieties without the intervention of a dirigent protein, a consequence of coniferyl alcohol accumulation or the dirigent protein retroinhibition as a result of the pinoresinol accumulation caused by the *LuPLR1* suppression.

Moreover, further change in secondary metabolites was also observed between LuT and Pi1AM lines in each plant part.

In roots, cyanogenic compounds such as linamarin and lotaustralin showed significantly higher contents in Pi1AM than those in LuT. While in stems, Pi1AM showed a lower content than LuT in linamarin and lotaustralin and a higher content in neolinustatin. On the other hand, in leaves, these cyanogenic compounds did not show any statistically significant changes, in the comparison between Pi1AM and LuT but linustatin, showed a higher content in Pi1AM line. A previous study on NMR metabolomics performed on flax seeds with different ω-3 fatty acid contents has shown that a decrease in secoisolariciresinol glucoside (+)-SDG was accompanied with a decrease in linustatin [33]. Another study on spatiotemporal distribution of lignans and cyanogenic glucosides in flaxseed revealed that seeds devoid of (+)-SDG did not accumulate linustatin [34]. However, so far, no specific and precise relation between lignans and cyanogenic compounds has been reported.

It should be noted that, although the majority of the metabolites listed above were measured by one of the two analytical techniques. The cyanogenic glycosides including linamarin, lotaustralin, linustatin, and neolinustatin, were detected in common with both ^1^H-NMR and LC-MS. Interestingly, for each of these metabolites, LC-MS data showed almost a similar relative value (Pi1AM/LuT), when compared to those obtained with ^1^H-NMR, in each of the plant parts. This similarity confirms the robustness of these two analytical methods. For example, for linamarin and lotaustralin: NMR data showed that Pi1AM/LuT root ratios were 1.44 and 1.5, respectively. LC-MS analysis, showed very similar values for Pi1AM/LuT ratios (1.41 for linamarin and 1.49 for lotaustralin). In stems, according to NMR data, Pi1AM/LuT ratio was 0.82 for linamarin and 0.88 for lotaustralin. While, using LC-MS, Pi1AM/LuT ratio were approximately 0.76 for linamarin and 0.76 for lotaustralin. In the case of leaves, both NMR and LC-MS data revealed no significant difference in lotaustralin and linamarin contents between Pi1AM and LuT.

In addition, in stems, Pi1AM showed a higher content of carlinoside, lucenin-2, orientin, caffeic acid glucoside (CAFG), chlorogenic acid, and ferulic acid glucoside (FAG) than in LuT. However, a lower content than LuT in triticuside-A.

Leaves of Pi1AM line showed a higher content in chlorogenic acid, carlinoside, FAG, lucenin-2 and CAFG, than shows the LuT. This latter showed, however, a higher content in vitexin and vicenin-2.

Carlinoside, lucenin-2, vitexin, vicenin-2, orientin and triticuside-A, are flavone C-glycosides derived from the same precursor of lignan, p-coumaroyl CoA, which in turn are derived from the phenylpropanoid pathway. Chalcone synthase (CHS) uses p-coumaroyl-CoA as a starter molecule to form the naringenin chalcone (4,2′,4′,6′-tetrahydroxychalcone), which serves as precursor for a different class of flavonoids [35]. Thus, the changes observed for these flavone C-glycosides in the transgenic line compared to the wild-type, could be explained by the disruption of their precursors p-coumaroyl CoA, which might be caused by the accumulation of coniferyl alcohol.

Ferulic and caffeic acids are also involved in phenylpropanoid metabolism. These compounds are hydroxylated derivatives of cinnamic acid. For monolignol biosynthesis, cinnamic acid is first hydroxylated by cinnamate 4-hydroxylase to yield *p*-coumaric acid. Then, *p*-coumaric acid and their derivates (caffeic and ferulic acid) are catalyzed by the action of 4-coumaroyl CoA ligase (4CL) to form the p-coumaroyl CoA ester, which is a point of divergence of different pathway leading to the biosynthesis of other phenylpropanoids.

The methylation of caffeoyl-CoA (activated form of caffeic acid) to feruloyl-CoA (activated form of ferulic acid) is catalyzed by caffeoyl-CoA *O*-methyltransferase. Then, feruloyl-CoA, is reduced by cinnamoyl-CoA reductase to form the coniferaldehyde and further by cinnamyl alcohol dehydrogenase to yield coniferyl alcohol [36].

In plant cells, ferulic and caffeic acids rarely appear in their free form. Generally, they are mostly found in glycosylated form, such as FAG and CAFG, or in esterified form, such as chlorogenic acid and chicoric acid [37]. The accumulation of these acids in glucoside and esterified forms in the transgenic line, can be due to the accumulation of coniferyl alcohol caused by the disruption of lignan synthesis. Thus, there might be a negative feedback on the activity of the coumaroyl CoA ligase (4CL), in order to reduce the biosynthesis of CoA esters which represent the activated forms of the metabolism of phenylpropanoids.

Based on the above mentioned, we can hypothesize that, the integration of PLR1-RNAi transgene in *L. usitatissimum* genome, affects flax metabolic activity and leads to a different metabolome in various plant parts of wild-type and transgenic lines.

#### 2.2.2. Metabolites Change in Response to Osmotic Stress in Wild-Type and PLR1-RNAi Transgenic Line

Drought is becoming one of the major limiting factors in agriculture worldwide leading to vast reductions in crop yield. It affects physiological and molecular processes and disrupt metabolic homeostasis, causing significant changes in the chemical composition of many plants resulting from the adjustment of metabolic pathways for adaptation. Hence, studying the metabolic response of transgenic line deprived for lignan production compared to wild-type of flax under osmotic stress conditions seems crucial and will provide important information about whether lignan play a role in the mechanism of the plants against this abiotic stress.

The PCA score plot of wild-type (LuT) and transgenic lines (Pi1AM) of each of the three parts (root, stem, and leaf), shows a characteristic grouping for control and stressed samples (Supplementary Data 3). The effect of the kinetics of stressed samples in different plant part was investigated. A Wilcoxon Rank Sum test was performed on the datasets for LuT and Pi1AM stressed sample, to verify whether there was a significant difference between the two periods of osmotic stress application. The test highlighted differences in metabolite content during the osmotic stress period (Data not shown). Thus, D1 and D5 stressed samples could be considered as a separate group for both lines.

##### Metabolic Profiles in Response to Osmotic Stress in Roots

**Figure 3:**
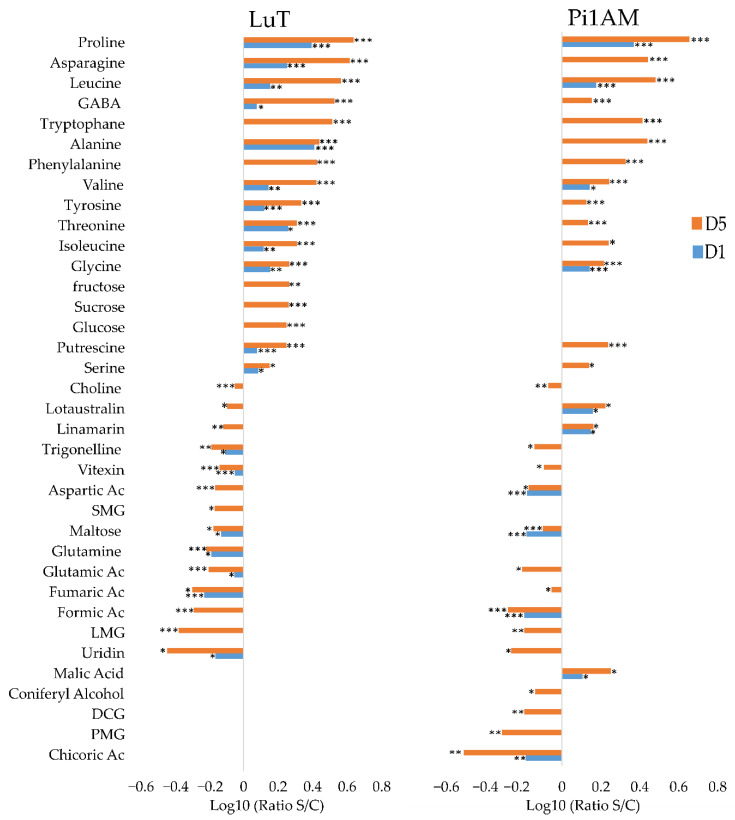
Comparison of metabolite content reorganization in wild-type (LuT) and transgenic (Pi1AM) roots after osmotic stress at different time-points: one and five days. Bars on logarithmic scale (log10) represent the mean relative response ratio S/C (content plant treated by PEG 6000/content control plant). Negative values represent a lower content and positive values a higher content of metabolites in stressed plants. Different time points are indicated by blue bars for D1 and red bars for D5. Values are significantly different at: *** *p* < 0.001; ** *p* < 0.01; * *p* < 0.05 after the Wilcoxon Rank Sum test (*n* = 8).

In roots, the metabolic response to osmotic stress differed between LuT and Pi1AM. Statistical analysis highlighted 31 metabolite contents that change under osmotic stress for LuT and 31 for Pi1AM (Figure 3). Among these, some responded similarly to osmotic stress in both LuT and Pi1AM, some were specific to LuT and others to Pi1AM.

Thirteen amino acid (AAs) that increased significantly during osmotic stress application period were common to both lines: proline, asparagine, leucine, GABA, tryptophane, alanine, phenylalanine, valine, tyrosine, threonine, isoleucine, glycine, and serine. The reorganization of almost all these AAs appeared after one-day post-treatment with PEG and becomes more visible after five days, which indicates that metabolite content variation in roots, occurred early in response to osmotic stress in both lines. The largest fold increase in AAs content was observed for proline. This compound accumulated in a gradual manner with stress period, and at D5, its content was multiplied by 4.99- and 4.52-fold (calculated by the formula: ratio S/C = content plant treated by PEG-6000/content control plant), for LuT and Pi1AM respectively. There was also an increase in the amount of putrescine, a diamine derived from amino acid, in stressed samples compared to control samples in both lines.

Decreased aspartic and glutamic acid contents were also observed in both lines, after PEG treatment. Only one AA changes specifically in LuT after PEG treatment: glutamine decreased in stressed roots samples compared to control samples in LuT, while the content of this metabolite was not affected in Pi1AM under osmotic stress.

In addition, LuT and Pi1AM showed different others kinds of primary metabolites that decreased during osmotic stress, such as choline, fumaric acid, formic acid, and uridine, as well as of secondary metabolites, such as trigonelline, vitexin, and LMG.

A significant accumulation of non-structural carbohydrates (NSCs) such as glucose (ratio S/C: 1.77), sucrose (ratio S/C: 1.82) and fructose (ratio S/C: 1.84), was observed specifically for LuT stressed samples, compared with LuT control samples, after five days of stress. Additionally, five days of treatment with PEG, led to a significant decrease in the content of secoisolariciresinol monoglucoside (SMG) (ratio S/C: 0.68) on LuT roots. However, this latter compound was not detected in Pi1AM roots.

For Pi1AM, five days of osmotic stress, induced a decrease in content of both lignans such as PMG (ratio S/C: 0.49) and neolignans such as DCG (ratio S/C: 0.64). A decrease in a similar manner was also observed for coniferyl alcohol content (ratio S/C: 0.7).

There were other metabolites that changed specifically in roots of Pi1AM, under osmotic stress. This was the case for chicoric acid that decreased and for malic acid that increased under osmotic stress conditions.

The content of linamarin and lotaustralin decreased in LuT, at D5 of stress conditions, with an S/C ratios of 0.8 and 0.76, respectively, while they accumulated more in Pi1AM in stress conditions with S/C ratios increased 1.45 and 1.67 folds respectively, after five days of osmotic stress, which was observed on both ^1^H-NMR and LC-MS.

##### Metabolic Profiles in Response to Osmotic Stress in Stems

**Figure 4:**
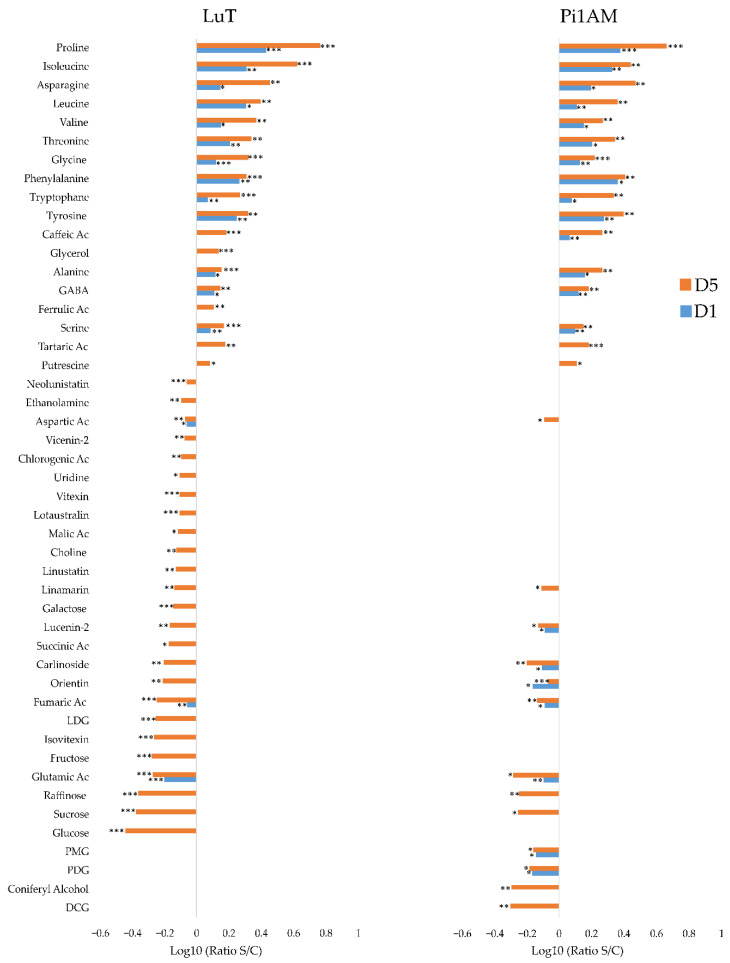
Comparison of metabolite content reorganization in wild-type (LuT) and transgenic (Pi1AM) stems after osmotic stress at different time-points: one and five days. Bars on logarithmic scale (log10) represent the mean relative response ratio S/C (content plant treated by PEG 6000/content control plant). Negative values represent a lower content and positive values a higher content of metabolites in stressed plants. Different time points are indicated by blue bars for D1 and red bars for D5. Values are significantly different at: *** *p* < 0.001; ** *p* < 0.01; * *p* < 0.05 after the Wilcoxon Rank Sum test (*n* = 8).

In stems, comparisons between the two datasets therefore reveal common responses between LuT and Pi1AM, and responses specific. A total of 43 metabolites were observed significantly influenced in the stressed LuT samples, compared to 29 in Pi1AM (Figure 4).

It appears that AAs content variation occurred early in stem of both lines (after one-day post treatment with PEG) relatively compared to other metabolites, and becomes more visible after five days post treatment with PEG.

Content of 12 among of these AAs increased (proline, isoleucine, asparagine, leucine, valine, threonine, glycine, phenylalanine, tryptophane, tyrosine, alanine, GABA, and serine), and for two of them it decreased (aspartic acid and glutamic acid). These reorganization in AAs content are similar to those observed in roots under osmotic stress. The highest increase also concerned the well-known osmorotectant proline that the content increased by 5.8- and 4.59-fold in the stems of LuT and Pi1AM, respectively, after five days of osmotic stress.

Other metabolites responded similarly to osmotic stress in the two lines. The contents of caffeic acid, tartaric acid and putrescine, were significantly accumulated under osmotic stress in both of the LuT and Pi1AM lines. In contrast, their contents in linamarin, lucenin-2, carlinoside, orientin, and fumaric acid were significantly decreased in stems, after osmotic stress.

The content of some kinds of sugar, including sucrose, and raffinose, was decreased in stems, for both Pi1AM and LuT lines, under osmotic stress.

Furthermore, glucose, fructose and galactose, decreased specifically in stems of LuT, after five days of osmotic stress.

The specific response to osmotic stress in LuT stems, was associated to the accumulation of ferulic acid and glycerol, and the decrease of the accumulation of primary metabolites such as succinic acid, choline, malic acid, uridine and ethanolamine, but also phenolic acid such as chlorogenic acid, flavonoids, such as isovitexin, vitexin, and vicenin-2, and cyanogenic glycosides, such as linustatin, lotaustralin, and neolinustatin, as well as lignans, such as LDG.

While the specific metabolic response to stress in Pi1AM, mainly consisted in a decrease of the content of metabolites involved in lignan biosynthetic pathway including DCG, coniferyl alcohol, PDG, and PMG.

##### Metabolic Profiles in Response to Osmotic Stress in Leaves

**Figure 5:**
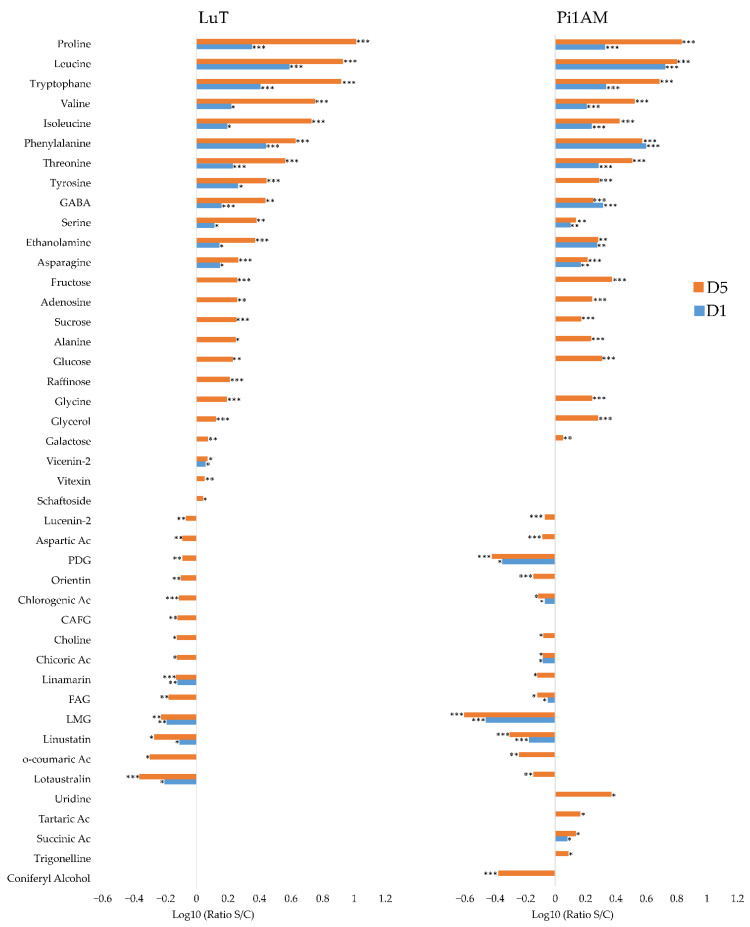
Comparison of metabolite content reorganization in wild-type (LuT) and transgenic (Pi1AM) leaves after osmotic stress at different time-points: one and five days. Bars on logarithmic scale (log10) represent the mean relative response ratio of S/C (content plant treated by PEG 6000/content control plant). Negative values represent a lower content and positive values a higher content of metabolites in stressed plants. Different time points are indicated by blue bars for D1 and red bars for D5. Values are significantly different at: *** *p* < 0.001; ** *p* < 0.01; * *p* < 0.05 after the Wilcoxon Rank Sum test (*n* = 8).

The response of metabolites to osmotic stress in leaves differed between LuT and Pi1AM lines. There were 38 metabolites with a significant change in leaves content under osmotic stress for each of LuT and Pi1AM (Figure 5). Most of up-regulated metabolites in both lines belonged to AAs, including proline, leucine, tryptophane, valine, isoleucine, phenylalanine, threonine, tyrosine, GABA, serine, asparagine, alanine, and glycine. In both lines it seems that the intensity of change of these AAs is dependent of stress period. The up-regulation had significantly been noticed after a single day of treatment with PEG and become more visible after five days. The largest significant increase in LuT and Pi1AM was proline with an S/C ratios of 10.29 and 6.8 respectively, after five days of osmotic stress. The AAs reorganization in leaves under osmotic stress is also reflected by the decrease in the content of aspartic acid in both lines. These trends in AAs changes are similar to what has been observed previously in roots and stems of both lines, under osmotic stress. A similar change in these AAs contents was reported by Quéro et al. in wild-type flax (*L. usitatissimum*) leaves, under osmotic stress conditions [38].

Other compounds accumulated in the same way in leaves of both lines when plants were submitted to osmotic stress. This was the case of ethanolamine, adenosine, and glycerol and as well as sugars, including fructose, sucrose, glucose, and galactose.

In addition to aspartic acid, there were 12 metabolites that the content decreased in leaves, under osmotic stress, which were common to both lines. This decrease was observed for lucenin-2, PDG, orientin, chlorogenic acid, choline, chicoric acid, FAG, LMG, and coumaric acid, but also for cyanogenic compounds including linamarin, linustatin, and lotaustralin. However, LuT showed specifically, significant increase of the accumulation of raffinose, vicenin-2, vitexin and schaftoside, and a decrease of the accumulation of CAFG in leaves under osmotic stress.

Conversely, some metabolites such as uridine, tartaric acid, succinic acid, trigonelline, and coniferyl alcohol were not affected by osmotic stress in LuT, but were significantly involved in the Pi1AM osmotic stress response. Among these metabolites, the content of coniferyl alcohol decreased while for the others, metabolites content in leaves increased under osmotic stress.

In the present work, an evident difference in the metabolic response to osmotic stress between different plant parts, as well as lines, was observed. These observations suggest that the mechanism of osmotic stress response of transgenic line is somehow different of that of wild-type, leading to the hypothesis that balanced lignan content may be important for proper stress response.

**Figure 6:**
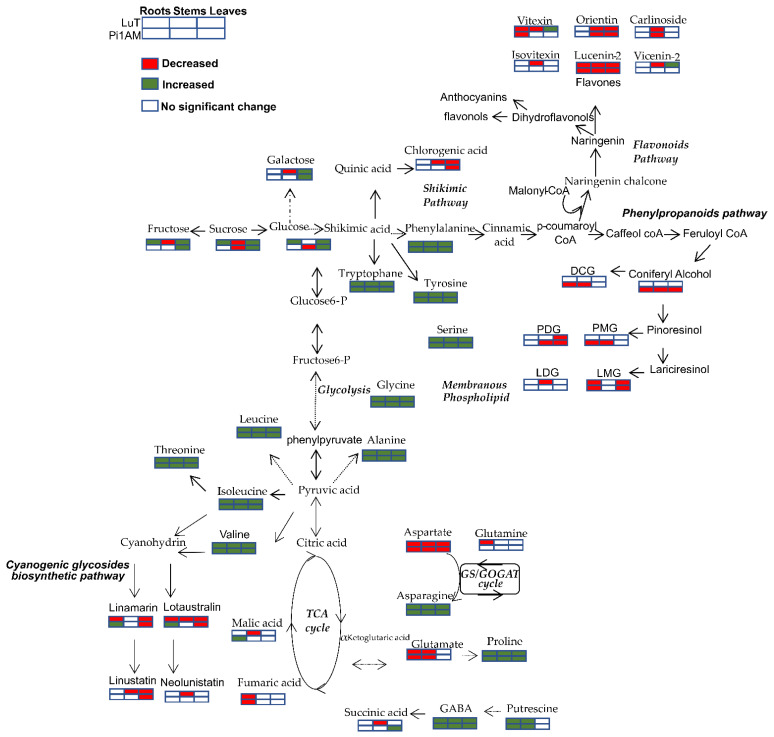
Major changes in the primary and secondary metabolic pathways of roots, stems and leaves of LuT and Pi1AM, after PEG treatment. The proposed metabolic pathways were based on the literature. Metabolites with red boxes represent significant decreases, with green ones represent significant increases, and with empty ones represent no significant change. The level of significance was set at *p* < 0.001, *p* < 0.01 and *p* < 0.05 after the Wilcoxon Rank Sum test.

Major changes in the primary and secondary metabolic pathways of roots, stems and leaves of both wild-type and transgenic line after PEG treatment, as well as the proposed relations between these metabolic pathways are presented in balance sheet shown in Figure 6.

#### 2.2.3. Comparison of the Metabolites in Wild-Type and PLR1-RNAi Transgenic Line under Osmotic Stress Conditions

**Figure 7:**
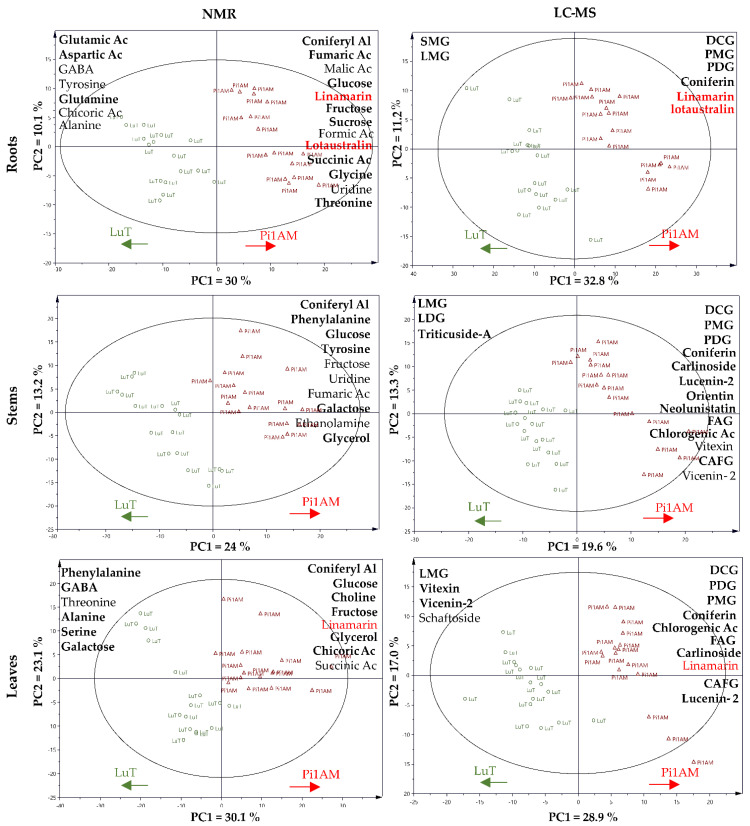
Score plot of partial least squares discriminant analysis (PLS-DA) of metabolite content in flax roots, stems and leaves, based on ^1^H-NMR and LC-MS data for LuT and Pi1AM, in stress conditions. Samples represent LuT plants (circle) and Pi1AM plants (triangle). Arrows indicate the increases in relative metabolite content in various parts of the wild-type (LuT) or transgenic (Pi1AM) plants. Metabolites are listed in descending order of discrimination. Boldface text indicates discriminant metabolites of the two lines in control and stress conditions. Red text indicates metabolites measured in common with LC-MS and ^1^H-NMR. Importance in Projection (VIP) score and the Log10 (ratio Pi1AM/LuT) of the main discriminant metabolites for each PLS-DA model, are shown in supplementary data 4.

PLS-DA was performed with both NMR and LC-MS data corresponding to stressed samples for LuT and Pi1AM for each of the three parts of the plant (Figure 7). The representation reveals that, in roots, stems, and leaves, LuT and Pi1AM lines are still separated because they still have a different metabolome under osmotic stress conditions. The metabolites that discriminated LuT and Pi1AM under osmotic stress in each of the three organs of these two lines, are represented in Figure 7, and their respective ratios, and significance are reported in Table 1.

In roots, some metabolites were discriminant of the two lines in control conditions and remained so, in the same range, in osmotic stress, due to a comparable increase or decrease in these metabolites after osmotic stress for both lines. This was the case for glutamic acid, aspartic acid, glutamine, SMG, and LMG that accumulated more in LuT, whereas glycine, succinic acid, threonine, fructose, sucrose, fumaric acid, glucose, lotaustralin, linamarin, coniferin, coniferyl alcohol, PDG, PMG, and DCG accumulated more in Pi1AM.

Some metabolites appeared to be discriminant for both lines only in stress conditions while they were not discriminant in control conditions. This was the case for GABA, tyrosine, alanine, uridine, formic acid, malic acid, chicoric acid, and trigonelline.

Some metabolites were discriminant for both lines in control conditions but no longer discriminant in stress conditions. This was the case for maltose, serine, and putrescine.

In stems, glycerol, galactose, tyrosine, glucose, phenylalanine, LMG, LDG, triticuside-A, CAFG, chlorogenic acid, FAG, neolinustatin, orientine, lucenin-2, carlinoside, DCG, PDG, PMG, coniferin, and coniferyl alcohol were discriminant of the two lines in control conditions and still discriminant under osmotic stress. All these metabolites are more accumulated in Pi1AM than in LuT, except LMG, LDG, and triticuside-A, which are accumulated more in LuT than in Pi1AM under stress conditions.

Ethanolamine, fumaric acid, uridine, fructose, vicenin-2 and vitexin appeared to be discriminant for both lines only in stress conditions. The amount of these metabolites was the same for both lines in control conditions:

Alanine, succinic acid, tartaric acid, asparagine, malic acid, putrescine, threonine, aspartic acid, glutamic acid, linamarin, and lotaustralin were discriminant for the two lines, in control conditions, but they did not interact in the discrimination of both lines, in stress conditions.

In leaves, some metabolites were discriminant of the two lines in both control and stress conditions. This was the case for phenylalanine, GABA, alanine, serine, galactose, LMG, vitexin, and vicenin-2 that were more accumulated in LuT and for glycerol, fructose, choline, glucose, lucenin-2, chicoric acid, CAFG, carlinoside, FAG, chlorogenic acid, coniferyl alcohol, coniferin, PMG, PDG, and DCG that were more accumulated in Pi1AM.

As for, threonine, succinic acid, schaftoside and linamarin, they appeared to be discriminant for LuT and Pi1AM, only in osmotic stress conditions. The amount of these metabolites was similar in LuT and Pi1AM, in control conditions.

In addition, uridine, adenosine, threonine, ethanolamine, asparagine, tartaric acid, raffinose, sucrose, tyrosine, and linustatin were discriminant for both lines, in control conditions whereas they are not discriminant in stress conditions.

Herein, osmotic stress appears to affect the whole plant metabolome in the different analyzed parts. Roots, stems, and leaves, display different responses in order to preserve water status for survival.

However, several organic molecules, known as osmolytes, play a crucial role during osmotic adjustment, including AAs, sugars, and organic acids, which potentially help in maintaining osmotic balance within the plant cells.

Amino acids protect the plants cell membranes, stabilize the structure of biomolecules, play a role as a scavenger of reactive oxygen species (ROS), and provide a reserve of nitrogen and carbon, mainly for the synthesis of specific enzymes and precursors for secondary metabolites such as flavonoids and lignins, during stress conditions [39,40,41]. Thus, an early accumulation of amino acids contributes to a greater level of stress tolerance [42].

In the present work, for the most of the amino acids that were measured, no significant differences in their concentrations have been reported between both lines, in control conditions. Furthermore, under osmotic stress, they showed a rapid transient increase in content, after one day of PEG treatment. This increase followed almost the same trend in LuT and Pi1AM lines.

Proline has been repeatedly reported as one of the most important components of osmotic adjustment in different plant species including flax [38,43]. A large variability exists between and within plant species for their capacity to accumulate proline [44]. Genotypes with high capacity to accumulate proline under osmotic stress are generally considered to be tolerant to this type of stress [45]. In this study, no significant differences in proline concentrations have been reported between both lines, in control conditions as well as after exposure to osmotic stress.

In particular, branched-chain amino acids (BCAAs), such as leucine, valine, and isoleucine, increase in response to osmotic stress, to enhance stress resistance. These compounds can serve as precursors for cyanogenic glycosides and other secondary metabolites, to acquire a defense response against abiotic stress [46]. In this study, the amount of leucine, valine and isoleucine, in roots, stems, and leaves, was the same for LuT and Pi1AM lines in control condition and remained so in stress conditions, due to a comparable increase in these metabolites during osmotic stress for both lines. We can thus assume that both LuT and Pi1AM showed the same capacity in producing AAs in their early response that enhances tolerance and/or resistance to osmotic stress, in various flax parts.

In addition to amino acid, the breakdown of homeostasis caused by water deficit induces the accumulation of carbohydrates. The accumulation of sugars mainly fructose and glucose, is indeed considered as an adaptive response that may also contribute to enhance plant stress tolerance [47]. Soluble sugar plays a major role in osmotic adjustment of plants facing the conditions of drought stress [48]. It also maintains the turgidity of leaves and prevent dehydration of membranes and proteins. Furthermore, sugar accumulation reduces leaf photosynthetic rate, under drought stress [49]. However, glucose enhances the plant adaptability under drought stress by inducing stomatal closure in leaves [50]. In another study, Quéro et al. have shown that glucose and fructose increased in response to osmotic stress in leaves of wild-type *L. usitatissimum* [38]. In this study, the glucose and fructose content of leaves increased significantly, in the same manner in both LuT and Pi1AM, in response to osmotic stress.

On the basis of the comparison of osmolyte contents between the two lines under control and osmotic stress, we may conclude that the transgenic line has a similar capacity with the wild-type line, in regulating osmotic stress by producing soluble sugars and AAs. Additionally, no visible differences were detected in terms of leaf area, plant height and plant dry weight in Pi1AM compared to LuT, when subjected to PEG-induced osmotic stress. Overall, it may be assumed that the capacity of plant to induce an adaptative response that improves osmotic stress tolerance was similar in both LuT and Pi1AM.

Moreover, our results showed that the levels of some secondary metabolites, including cyanogen, lignan, and flavonoids, differ between wild-type and transgenic plants in both control and stress conditions.

Interestingly, a significant common decrease in the content of metabolites involved in lignan biosynthetic pathway was observed at least in one part of plants subjected to osmotic stress in comparison to control plants. This was the case for coniferyl alcohol, PMG and DCG that decreased specifically in Pi1AM, for LMG that decreased in both lines, for SMG and LDG that decreased only in LuT, and for PDG that decreased in both lines in leaves, but only in Pi1AM in stems, under stress conditions. These results indicate that osmotic stress response and lignan biosynthesis pathways may have a crosstalk with each other in *L. usitatissimum*.

## 3. Materials and Methods

### 3.1. Plant Material

Flax seeds of the variety Barbara, for PLR1-RNAi transgenic (Pi1AM, homozygous), and wild-type (LuT) lines, have been provided by the Laboratory of Woody Plants and Crops Biology (LBLGC, INRAE USC 1328) in Chartres, France. Plants were grown in a hydroponic cultivation system on Hoagland solution [51,52], in a growth chamber with a hemeroperiod of 16 h at 21 °C, 70% relative humidity and a light intensity of 90 µmol∙m^−1^·s^−1^. After 30 days, half of each line was grown on Hoagland medium supplemented with PEG-6000 (30 mM) (stressed plants), while the half remaining plants still grown on Hoagland medium (control plants). Then, control and treated plants were harvested at different time-points: one and five days (D1, D5) after osmotic stress application. During the harvest, the aerial and root parts from each plant, were separated, immersed in liquid nitrogen, stored at −80 °C, and freeze-dried. Leaves, stems, and roots of each plant, were then separately ground to a fine powder. For each condition, eight independent samples coming from different plants have been collected (*n* = 8) and were subjected to further analysis.

### 3.2. PCR Analysis of Transgenic Plants

Genomic DNA was extracted from leaves (10 mg dried weight) of wild-type and transgenic plants, using the E.Z.N.A.^®^ Plant DNA DS Kit (Omega, Bio-teK (Norcross, GA USA)), according to the manufacturer’s instructions, and quantified using a Nanodrop^®^ spectrophotometer. To confirm their transgenic nature, the extracted DNA from all isolates were tested for the presence of *NPTII* selectable marker gene. Primers used for the amplification of a 0.7-Kb *NPTII* fragment were: P2, 5′-ATCGGGAGCGGCGATACCGTA-3′ (position 201) as 5′ primer and P1, 5′-GAGGCTATTCGGCTATGA CTG-3′ (position 900) as 3′ primer [25]. PCR amplification was carried out in a total volume of 25 μL containing 12.5 μL of Quick-Load^®^ Taq 2X Master Mix, BioLabs, 0.5 μL of each primer (0.25 μM), 2 µL of extracted DNA and 10 µl ultrapure water. Amplifications were performed with initial denaturation step of 5 min at 95 °C, followed by 40 cycles of 10 s denaturation at 95 °C, 30 s annealing at 55 °C for the *NPTII* gene, and a final extension step of 1 min at 68 °C. PCR products were run in 2% agarose gel electrophoresis, with 0.5X TBE (Tris, borate, EDTA), at 100 V for 45 min, and bands were visualized with GelRed, under UV light, using a ChemiDoc Imaging system.

### 3.3. Metabolite Wxtractions

Metabolites were separately extracted from 100 mg of powdered leaves or stems and 60 mg of powdered roots. A total volume of 800, 700 and 600 µL Water/methanol (1:1), used as extracting solvent, was added to leaves, stems and root powder respectively, and the samples were mixed for 10 min at 60 °C, using a ThermoMixer^®^ (Eppendorf AG, Germany) at 2000 rpm, followed by 30 min of sonication at 60 °C using ultrasonic bath at 35 kHz. The samples were centrifuged at 4 °C for 10 min at 12,000 rpm. A total of 400 µL of the supernatant were collected. The described extraction process was repeated two times by adding 400 µL of extraction solvent in the beginning of each new extraction. A final volume of 1.2 mL was collected, of which 800µL were used for NMR analysis. The remaining 400 µL were then used for LC-MS analysis.

### 3.4. Metabolite Analysis by NMR

#### 3.4.1. Sample Preparation

The pH of each sample prepared for NMR analysis was adjusted to 6.00 ± 0.02, and the samples were dried under vacuum and then dissolved in 800 µL of deuterated solvent prepared in a mixture of (1:1) Methanol-d4: KH_2_PO_4_ buffer (0.1 M) in D_2_O at pH 6.0 with TMSP (0.0125%), NaN_3_ (0.6 mg/mL), and 15 mM EDTA-d12. Then, samples were briefly vortexed, sonicated, and centrifuged. The supernatant was placed in 5-mm NMR tubes and then used for NMR analysis.

#### 3.4.2. NMR Data Acquisition

All NMR spectra were acquired at 300 K with a Bruker Avance III 600 MHz spectrometer operating at 600.13 MHz for ^1^H, and 150.91 MHz for ^13^C, using a 5-mm multinuclear broadband, equipped with z-gradient (TXI 5 mm tube). CD_3_OD was used as the internal lock. All 1D ^1^H-NMR spectra were collected using 32 scans of 131 K data points and a spectral width of 8417 Hz with a relaxation delay of 13 s, and a water suppression pulse sequence. The resulting spectra were automatically phased and baseline corrected, using Bruker Topspin software (version 3.5) [51]. 2D J-resolved NMR spectra were processed with a 2 s relaxation delay using 16 scans per 64 increments collected into 64K data points, with spectral widths of 8417.5 Hz in F2 and 50 Hz in F1. 2D HSQC spectra were recorded with a 2 s relaxation delay, using 64 scans per 256 increments that were collected into 4 K data points using spectral widths of 8417.5 Hz in F2 and 50 Hz in F1. All spectra were calibrated to TMSP at 0.0 ppm by the Topspin v3.5 (Bruker) software. Each of the 2 D acquisitions were performed by analyzing one control and one stressed samples of each part of plant corresponding to each of the two lines (Pi1AM and LuT).

#### 3.4.3. NMR Data Treatment

^1^H-NMR spectra were automatically converted into ASCII format and the data from each part of plant (roots, stems and leaves) were collected and imported into Matlab software (version 2018a, the Mathworks Inc, Natick, MA, USA) where baseline correction was performed with the algorithm “airPLS 2.0” [53,54,55]. All spectra were aligned using the icoshift algorithm (v 1.2.1) with manually defined alignment bins. Then specific integration intervals of the spectra ‘‘buckets’’ were defined manually, and each bucket was integrated. Regions of the NMR spectra corresponding to the methanol-d4 (3.34–3.30 ppm), to residual water (4.85–4.70 ppm), to TMSP (0.01–0 ppm) and to PEG-6000 (3.68–3.64 ppm), were removed. All signals with intensities lower than 3.3 times the mean variance from such a noise region were considered to be noise and were also removed. The obtained datasets were then used for statistical analysis. Metabolites in the 1D and 2D NMR spectra of flax extracts were identified based on comparison with spectra and chemical shifts of reference compounds of database previously developed in the laboratory.

#### 3.4.4. Metabolite Analysis by LC/MS

##### Sample Preparation

Extracts of stems and leaves were diluted 5 and 10 times, respectively, with methanol/water (50/50), while no dilution was performed for root extracts. All samples were filtered through 0.22 µm PTFE membrane filters and placed in glass vials for further LC-MS analysis. For each plant part, a QC sample were prepared; 10 µL from 20 different samples were taken and thoroughly mixed, reaching a total volume of 200 µL. For roots, stems, and leaves, analysis samples were run in a randomized order.

##### LC-MS Data Acquisition

The metabolomics analysis was performed using ACQUITY UPLC H-Class system (Waters Micromass, UK), coupled to a SYNAPT G2-Si Q-TOF mass spectrometer (Waters-Micromass, Manchester, UK), which was equipped with an electrospray ion source (ESI). UPLC separation was performed using a Kinetex C18 (1.7 µm, 100 mm × 2.1 mm, Phenomenex, Torrance, CA, USA) column. The column temperature was maintained at 50 °C. The injected volume was 1 µL. Water (A) and methanol (B), both supplemented with 0.1% formic acid, were used as mobile phases. A stepwise gradient method was used for elution at a flow rate of 0.4 mL/min, with the following conditions: 5–95% B (0–7 min), followed by 3 min of isocratic 95% solvent B, and 1 min gradient to 95% (A), followed by 5 min of re-equilibrium at 100% A. MS data was collected in the negative ion mode, over a m/z range of 50 to 1150. The parameters of electrospray ionization (ESI) source were set as follows: capillary voltage at 3 keV, the cone voltage at 3 V, source temperature at 120 °C, desolvation at 450 °C, the cone gas flow 6.5 bar and the desolvation gas flow 800 L/h. Analyses of the samples were carried out in a mode of a full MS survey, at a resolution of 2000 (FWHM). MSMS scans for most intense peaks were performed to produce high resolution MSMS spectra, with a collision energy of 30 eV. Data acquisition was performed by MasslynxTM v4.1 software (Waters, Milford, MA, USA). The QC samples were injected at the beginning of the run to set up the system and then every eight samples, so they were used to ensure system conditioning within the analytical sequence.

##### LC-MS/MS Data Processing

The acquired spectral data were converted to mzXML format using the Proteowizard MSConvert tool from 0 to 10 min RT in order to avoid features coming from cleaning step of the gradient.

Then, the mzXML files data of each part of the plant were loaded and pre-processed with the XCMS package (v3.0.2) in the open-source R software (v3.2.2).

The centWave algorithm was used for peak detection with the following optimized parameters: minimum peak width = 3 s, maximum peak width = 15 s, ppm = 5, threshold = 2, mzdiff = 0.005 and prefilter: (4,100,000), and noise filter = 1000. Peaks were well aligned by XCMS, using the following parameters: bw = 5.0 and mzwid = 0.025. Retention time correction was performed by the Obiwarp algorithm, which aligns multiple samples by using a center sample.

A filling step was included to reduce the number of missing peaks, using the fillPeaks tool. For each sample, Peak area, retention time and peak widths, calculated as the difference between the end and start of the integration points, were extracted from XCMS data.

After the whole data processing, a table (matrix) including retention time and m/z, sample names, and ion intensities, was obtained for each sample set. Then, before the statistical analysis, these matrices were prepared in order to remove the features before the injection peak (less than 1 min).

The percentage of relative standard deviation (%RSD) was calculated for all metabolic features in QC samples and the features with %RSD greater than 30% were removed due to its variability.

Metabolites were principally identified by matching masses, retention times and fragment patterns of pure standards.

##### Statistical Treatment of Data

The obtained datasets for NMR and LC-MS were imported into SIMCA-P software (version 15.0; Umetrics, Umea, Sweden), and applied to principal component analysis (PCA) and partial least squares discriminant analysis (PLS-DA) for multivariate statistical analysis. The UV scaling method was used.

The Wilcoxon Rank Sum test was used in R’s statistics base-package in order to test the significant difference in metabolite content, between analyzed groups, with different *p*-values (*p* < 0.05; *p* < 0.01; *p* < 0.001).

## 4. Conclusions

In the present study, the wild-type and transgenic flax plants displayed different metabolic behaviors after being subjected to osmotic stress conditions, which could be due to their different metabolic backgrounds but also to different metabolic rearrangements. In the last case, we can suggest that an altered lignan composition in transgenic plant changes the metabolic response of the plant to osmotic stress.

In addition, our experimental observations revealed that wild-type and transgenic line showed the same morphological response to osmotic stress. Moreover, they showed the same capacity to induce an adaptive response that improves osmotic stress tolerance. Thus, we can assume that lignan perturbations did not affect plant stress response leading to osmotic stress resistance. Taken together, our findings indicate that the lignans could not be directly involved in osmotic stress resistance, but in the metabolic pathways responsible of the osmotic stress response.

## Figures and Tables

**Table 1:** Comparison of discriminant metabolite contents for LuT and Pi1AM in stress versus control conditions.

Roots			Stems			Leaves		
	Ratio Pi1AM/LuT			Ratio Pi1AM/LuT			Ratio Pi1AM/LuT	
Metabolite	Stress	Control	Metabolite	Stress	Control	Metabolite	Stress	Control
**Primary metabolites**			**Primary metabolites**			**Primary metabolites**		
Glutamic Acid	0.5 ***	0.65 **	Glycerol	1.3 *	5.56 **	Phenylalanine	0.42 **	0.7 **
Aspartic Acid	0.53 ***	0.55 ***	Ethanolamine	1.38 **	_	GABA	0.47 *	0.63 *
GABA	0.53 ***	_	Galactose	1.47 *	1.19 **	Threonine	0.47 **	_
Tyrosine	0.54 ***	_	Fumaric Acid	1.5 **	_	Alanine	0.62 *	0.64 ***
Glutamine	0.56 ***	0.63 ***	Uridine	1.68 **	_	Serine	0.64 *	1.13 *
Alanine	0.85 ***	_	Fructose	1.87 *	_	Galactose	0.66 **	0.75 ***
Threonine	1.2 ***	1.23 *	Tyrosine	2.01 ***	1.88 ***	Succinic Acid	1.2 *	_
Uridine	1.34 ***	_	Glucose	2.31 **	1.42 **	Glycerol	1.36 *	1.5 **
Glycine	1.38 ***	1.41 ***	Phenylalanine	3.05 ***	1.6 **	Fructose	2.00 *	1.72 ***
Succinic Acid	1.42 ***	1.64 **	Coniferyl Alcohol	7.72 ***	8.65 ***	Choline	2.05 **	1.75 ***
Formic Acid	1.43 ***	_	Alanine	_	0.7 **	Glucose	2.15 **	1.34 *
Sucrose	1.45 ***	2.07 ***	Succinic Acid	_	0.73 ***	Coniferyl Alcohol	4.79 ***	10.32 ***
Fructose	1.69 ***	1.73 ***	Tartaric Acid	_	0.74 **	Uridine	_	0.55 *
Glucose	1.79 ***	1.85 ***	Asparagine	_	0.76 *	Adenosine	_	0.6 ***
Malic Acid	2.03 ***	_	Malic Acid	_	0.82 ***	Threonine	_	0.76 **
Fumaric Acid	2.11 ***	1.34 *	Putrescine	_	0.84 **	Ethanolamine	_	0.79 **
Coniferyl Alcohol	5.51 ***	7.91 ***	Threonine	_	0.87 *	Asparagine	_	0.85 *
Maltose	_	0.87 *	Aspartic Acid	_	1.27 *	Tartaric Acid	_	0.91 *
Serine	_	1.48 ***	Glutamic Acid	_	1.39 *	Raffinose	_	1.28 **
Putrescine	_	1.6 ***	**Secondary metabolites**			Sucrose	_	1.32 **
**Secondary metabolites**			LMG	0.46 ***	0.73 ***	Tyrosine	_	3.73 ***
SMG	0.06 ***	0.15 ***	LDG	0.64 ***	0.17 ***	**Secondary metabolites**		
LMG	0.28 ***	0.31 **	Triticuside-A	0.8 *	0.8 **	LMG	0.27 ***	0.50 ***
Chicoric Acid	0.71 ***	_	Vicenin-2	1.36 ***	_	Vitexin	0.65 ***	0.69 ***
Trigonelline	1.21 ***	_	CAFG	1.45 **	1.42 **	Vicenin-2	0.7 **	0.77**
Lotaustralin	1.81 **	1.49 **	Vitexin	1.54 ***	_	Schaftoside	0.9 ***	_
Linamarin	1.75 **	1.41 **	Chlorogenic Acid	1.63 **	1.32 **	Lucenin-2	1.27*	1.21 **
Coniferin	1.83 ***	2.32 ***	FAG	1.81 ***	1.21 *	Chicoric Ac	1.33 ***	1.23 ***
PDG	12.66 ***	12.27 ***	Neolunistatin	2.04 **	1.84 **	CAFG	1.42 *	1.11 **
PMG	18.63 ***	18.13 ***	Orientin	2.67 ***	1.92 ***	linamarin	1.45 ***	_
			Lucenin-2	3.07 ***	2.57 ***	Carlinoide	1.5 ***	1.45 ***
			Carlinoside	3.51 **	2.8 ***	FAG	1.66 ***	1.24 *
			Coniferin	3.63 ***	2.4 ***	Chlorogenic Acid	2.19 ***	1.51 **
			PDG	3.83 ***	4.73 ***	Coniferin	6.45	7.68
			PMG	4.25 ***	5.31 ***	PMG	14.1	13.78 ***
			Linamarin	_	0.76 ***	PDG	14.5 ***	15.03 ***
			Lotaustralin	_	0.76*	Linustatin	_	1.27 **

Note: Results of the Wilcoxon Rank Sum test and ratios are reported for each discriminant metabolite; Values are significantly different at: *** *p* ˂ 0.001; ** *p* ˂ 0.01; * *p* ˂ 0.05. After the Wilcoxon Rank Sum test.

## Data Availability

The data presented in this study are available on request from the corresponding author.

## Supplementary Materials

The following are available online, Table S1. Comparison of dry weight of different part (roots, stems and leaves) of wild-type (LuT) and transgenic (Pi1AM) flax lines, at D1 and D5 in control conditions (*n* = 8). Table S2. The VIP score and Log10 (Ratio Pi1AM/LuT) of the discriminant metabolites between LuT and Pi1AM of different plant part, in control conditions. Figure S1. Score plot of principal component analysis (PCA) based on ^1^H-NMR and LC-MS data for LuT control or stressed samples in flax roots, stems and leaves. Figure S2. Score plot of principal component analysis (PCA) based on 1H-NMR and LC-MS data for Pi1AM control or stressed samples in flax roots, stems and leaves. Table S3. The VIP score and Log10 (Ratio Pi1AM/LuT) of the discriminant metabolites between LuT and Pi1AM of different plant part, in stress conditions.

## Sample Availability

Samples of the compounds are available on request from the corresponding author.
