# Supplementary material for: NMR and LC-MS-Based Metabolomics to Study Osmotic Stress in Lignan-Deficient Flax
Source: Molecules. 2021 Feb 2;26(3):767. doi: 10.3390/molecules26030767 (PMC7867241; doi:10.3390/molecules26030767)
Supplement: Supplementary file 1 [file molecules-26-00767-s001.pdf]

Table S1. Comparaison of dry weight of different part (roots. stems and leaves) of wild-type (LuT) and transgenic (Pi1AM) flax lines, at D1 and D5 in control conditions (n=8).

		Dry Weight (mg)															
		Pi1AM								*LuT*							
	N°	1	2	3	4	5	6	7	8	1	2	3	4	5	6	7	8
**Roots**	D1	147.9	158.7	106.8	229.57	168.8	116.4	209.1	125.3	141.3	154.1	158.3	112.2	144.6	156.8	173.3	142
	D5	133.8	123	117.4	128	148.3	146.9	178.6	156.5	114.7	117.7	118.9	157.7	150.4	185.9	159.3	102.9
**Stems**	D1	563.90	688.40	483.10	653.60	476.60	587.50	494.50	460.90	517.40	632.20	576.50	685.80	524.40	599.40	695.90	658.10
	D5	907.70	787.00	869.50	834.50	875.90	788.20	825.60	931.80	784.00	704.60	738.70	681.40	969.90	861.40	889.90	688.50
**Leaves**	D1	567.78	650.60	533.60	547.19	639.07	555.28	540.81	436.63	475.83	574.35	636.72	594.95	470.78	452.18	664.36	561.73
	D5	841.42	978.52	846.79	676.91	861.23	713.56	843.6	993.09	675.15	731.79	66.14	653.18	820.81	813.48	889.46	890.4

Table S2. The VIP score and Log10 (Ration Pi1AM/LuT) of the discriminant metabolites between LuT and Pi1AM of different plant part, in control conditions.

	**Roots**	**VIP score**	**Log10 (Ratio Pi1AM/LuT)**
**LC-MS**	DCG	1.89019	-
	PMG	1.81812	1.25
	PDG	1.51188	1.08
	Coniferin	1.20745	0.36
	lotaustralin	0.89665	0.17
	Linamarin	0.856045	0.14
	SMG	1.84952	-0.82
	LMG	1.73832	-0.5
**NMR**	Coniferyl Alcohol	1.31626	0.89
	Sucrose	1.01421	0.31
	Glucose	1.00465	0.26
	Fructose	0.92145	0.23
	Succinic Acid	0.90235	0.21
	Putrescine	0.90565	0.20
	Serine	0.86594	0.17
	Lotaustralin	0.85335	0.17
	Linamarin	0.866358	0.15
	Glycine	0.844595	0.14
	Fumaric Acid	0.832954	0.12
	Threonine	0.790184	0.08
	Aspartic Acid	1.09956	-0.25
	Glutamine	1.056587	-0.2
	Glutamic Acid	1.005789	-0.18
	Maltose	0.75862	-0.06

	**Stems**	**VIP score**	**Log10 (Ratio Pi1AM/LuT)**
**LC-MS**	DCG	2.27601	-
	PMG	2.11299	0.72
	PDG	1.85297	0.67
	Carlinoside	1.58547	0.44
	Lucenin-2	1.55471	0.43
	Coniferin	1.4326	0.38
	Orientin	1.2658	0.28
	Neolunistatin	1.09578	0.26
	CAFG	0.8868	0.15
	Chlorogenic Acid	0.8258	0.12
	FAG	0.8164	0.08
	LDG	2.14607	-0.76
	LMG	1.45473	-0.13
	Linamarin	1.00458	-0.11
	lotaustralin	1.00546	-0.11
	Triticuside-A	1.00541	-0.09
**NMR**	coniferyl Alcohol	1.84679	0.93
	Glycerol	1.25878	0.74
	Tyrosine	1.15689	0.27
	Phenylalanine	1.14565	0.2
	Glucose	1.01569	0.15
	Glutamic Acid	0.90458	0.14
	Aspartic Acid	0.90469	0.10
	Galactose	0.78698	0.07
	Alanine	0.90125	-0.15
	Succinic Acid	0.88985	-0.13
	Tartaric Acid	0.88875	-0.13
	Asparagine	0.81564	-0.11
	Malic Acid	0.76594	-0.08
	Linamarin	0.71568	-0.08
	Putrescine	0.71238	-0.07
	Threonine	0.70023	-0.06
	Lotaustralin	0.65892	-0.05

	Leaves	VIP score	Log10 (Ratio Pi1AM/LuT)
LC-MS	DCG	1.90774	-
	PDG	1.87959	1.17
	PMG	1.81343	1.13
	Coniferin	1.41243	0.88
	Chlorogenic Acid	1.00678	0.17
	Carlinoside	1.05864	0.16
	Linustatin	0.85687	0.1
	FAG	0.76987	0.09
	Lucenin -2	0.69658	0.08
	CAFG	0.68659	0.04
	LMG	1.495326	-0.3
	Vitexin	0.89178	-0.16
	Triticuside-A	0.79576	-0.11
NMR	coniferyl Alcohol	1.61418	1.01
	Tyrosine	1.05687	0.57
	Choline	1.09456	0.24
	Fructose	1.01867	0.23
	Glycerol	1.00356	0.17
	Glucose	1.00238	0.12
	Sucrose	1.00568	0.12
	Raffinose	0.88657	0.1
	Chicoric Acid	0.76125	0.08
	Serine	0.76575	0.05
	Uridine	1.01567	-0.25
	Adenosine	1.00458	-0.22
	GABA	0.90254	-0.2
	Alanine	0.87869	-0.19
	Phenylalanine	0.80051	-0.15
	Galactose	0.79476	-0.12
	Threonine	0.78697	-0.11
	Ethanolamine	0.74567	-0.1
	Asparagine	0.70865	-0.07
	Tartaric Acid	0.70681	-0.04

Supplementary data 3

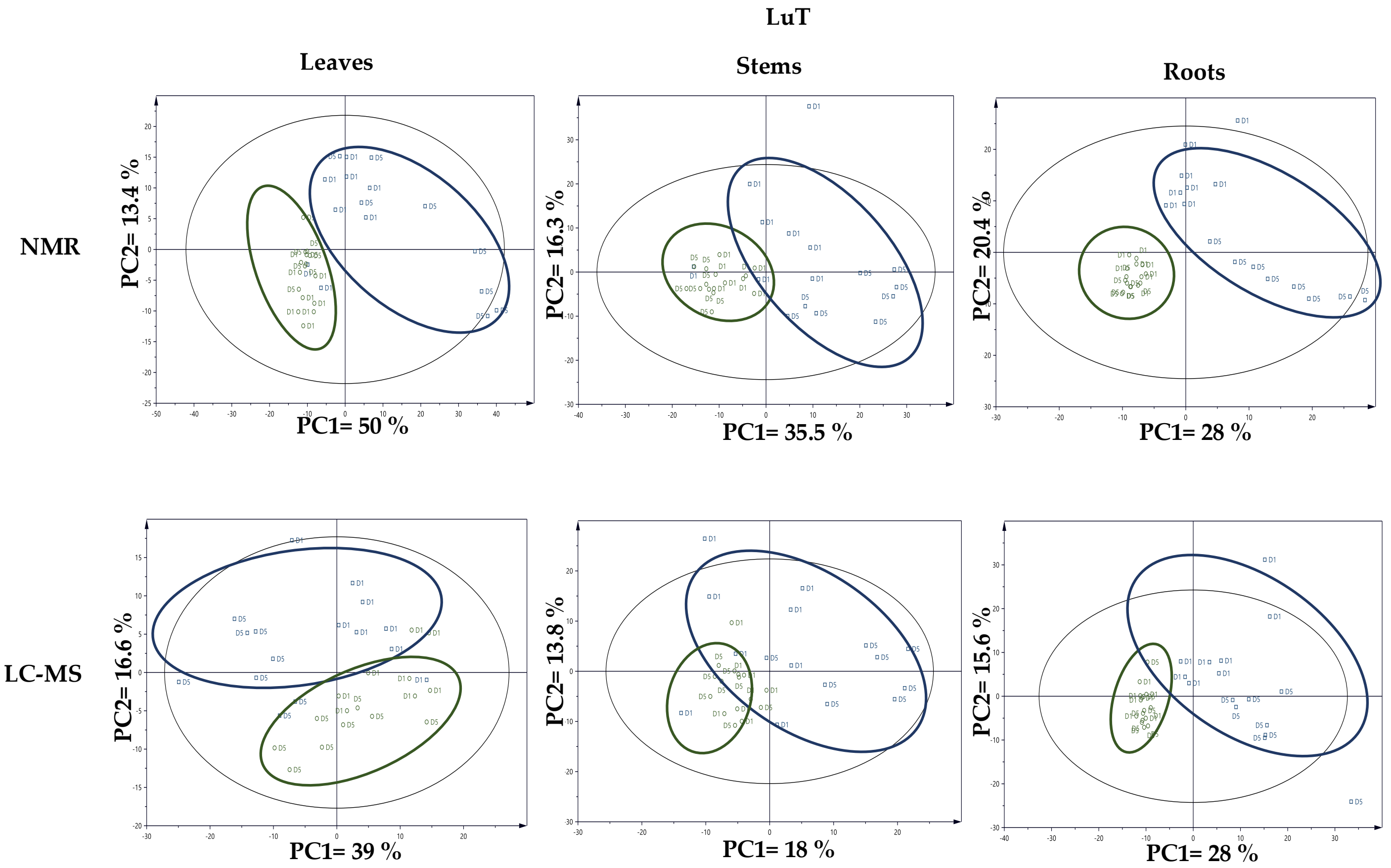

Figure S1. Score plot of principal component analysis (PCA) based on ^{1}H-NMR and LC-MS data for LuT control or stressed samples in flax roots, stems and leaves.

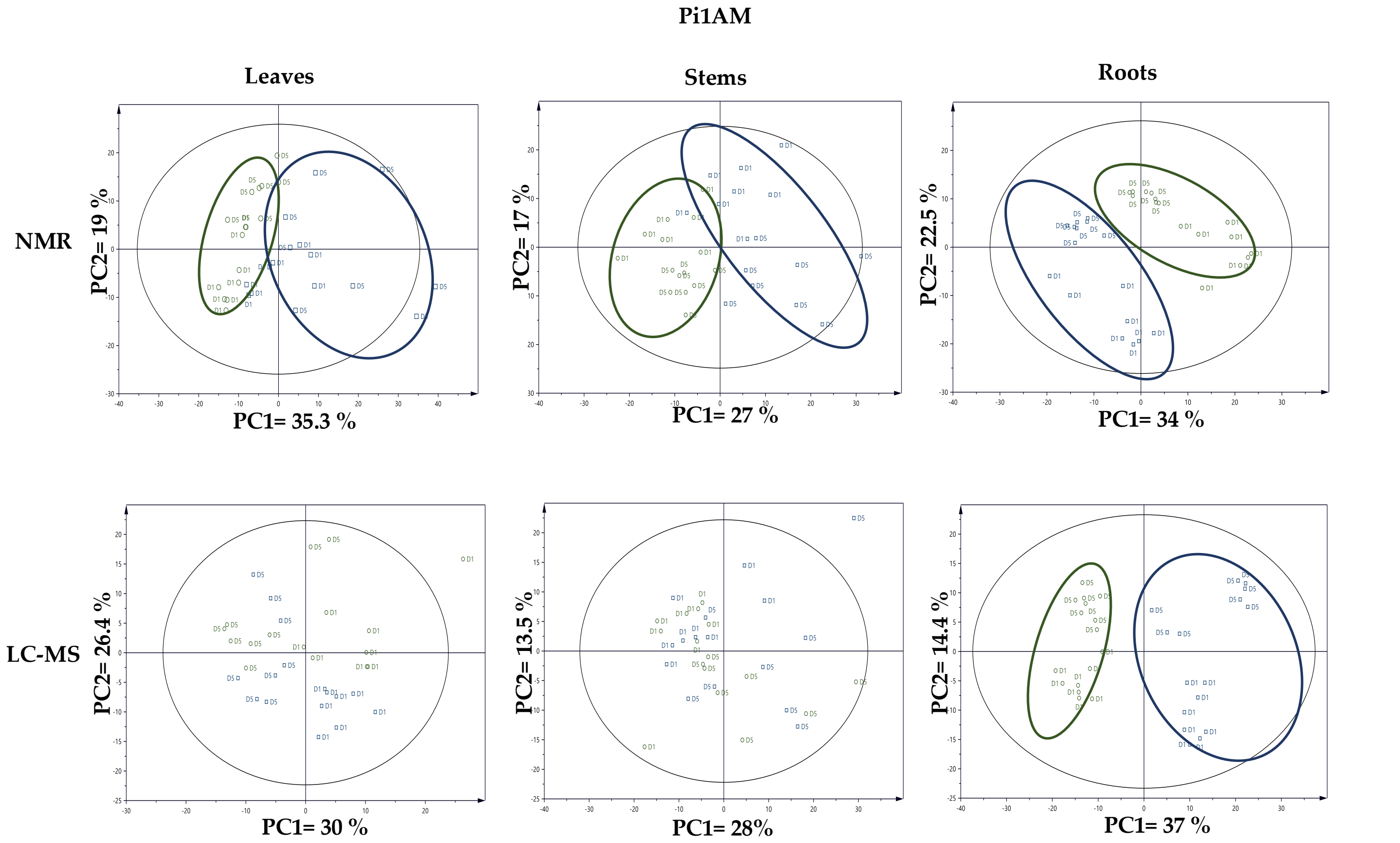

Figure S2. Score plot of principal component analysis (PCA) based on ^{1}H-NMR and LC-MS data for Pi1AM control or stressed samples in flax roots, stems and leaves.

Table S3. The VIP score and Log10 (Ration Pi1AM/LuT) of the discriminant metabolites between LuT and Pi1AM of different plant part, in stress conditions.

	**Roots**	**VIP score**	**Log10 (Ratio Pi1AM/LuT)**
**LC-MS**	DCG	1.92485	-
	PMG	1.49995	1.27
	PDG	1.35502	1.1
	Coniferin	1.10447	0.26
	Linamarin	0.97165	0.24
	Lotaustralin	0.95045	0.17
	SMG	1.87131	-1.22
	LMG	1.455905	-0.55
**NMR**	Coniferyl Al	1.34104	0.74
	Fumaric Acid	1.0525	0.32
	Malic Acid	1.01368	0.30
	Glucose	0.8586	0.25
	Linamarin	0.85165	0.24
	Fructose	0.82681	0.22
	Sucrose	0.74568	0.16
	Formic Acid	0.68257	0.15
	Lotaustralin	0.659256	0.15
	Succinic Acid	0.66465	0.15
	Glycine	0.60701	0.13
	Uridine	0.60254	0.12
	Threonine	0.55691	0.07
	Glutamic Acid	1.0125	-0.3
	Aspartic Acid	0.90697	-0.27
	GABA	0.79855	-0.27
	Tyrosine	0.74521	-0.26
	Glutamine	0.76524	-0.25
	Chicoric Acid	0.70685	-0.14
	Alanine	0.69252	-0.07

	Stems	VIP score	Log10 (Ratio Pi1AM/LuT)
LC-MS	DCG	2.1672	-
	PMG	1.96605	0.62
	PDG	1.5767	0.58
	Coniferin	1.49128	0.55
	Carlinoside	1.4875	0.54
	Lucenin-2	1.39651	0.48
	Orientin	1.32568	0.42
	Neolunistatin	1.01352	0.3
	FAG	1.00635	0.25
	Chlorogenic Acid	0.96665	0.21
	Vitexin	0.902365	0.18
	CAFG	0.901123	0.16
	Vicenin-2	0.90658	0.13
	LMG	2.40246	-0.33
	LDG	1.97926	-0.19
	Triticuside-A	0.8524	-0.09
NMR	Coniferyl Al	1.7112	0.88
	Phenylalanine	1.2135	0.48
	Glucose	1.0536	0.36
	Tyrosine	1.0235	0.3
	Fructose	1.0004	0.27
	Uridine	0.9591	0.21
	Fumaric Acid	0.9135	0.17
	Galactose	0.9069	0.16
	Ethanolamine	0.84231	0.13
	Glycerol	0.78654	0.11

	Leaves	VIP score	Log10 (Ratio Pi1AM/LuT)
**LC-MS**	DCG	1.95681	-
	PDG	1.35502	1.16
	PMG	1.28474	1.14
	Coniferin	1.082656	0.8
	Chlorogenic Acid	0.8958	0.34
	FAG	0.86235	0.22
	Carlinoside	0.81658	0.17
	Linamarin	0.80658	0.16
	CAFG	0.80569	0.15
	Lucenin-2	0.75052	0.1
	LMG	1.499468	-0.56
	Vitexin	0.90695	-0.18
	Vicenin-2	0.90556	-0.15
	Schaftoside	0.70581	-0.045
**NMR**	Coniferyl Al	1.74776	0.68
	Glucose	1.09261	0.33
	Choline	1.0569	0.31
	Fructose	1.02364	0.3
	Linamarin	0.91756	0.15
	Glycerol	0.90235	0.13
	Chicoric Acid	0.8654	0.12
	Succinic Acid	0.80231	0.079
	Phenylalanine	1.0235	-0.37
	GABA	1.00568	-0.32
	Threonine	1.00128	-0.32
	Alanine	0.9123	-0.2
	Serine	0.8568	-0.19
	Galactose	0.8165	-0.18
